# Histological and Ultrastructural Description of Benign Adipocytic Tumors in Farmed Striped Sea Bream (*Lythognathus mormyrus*)

**DOI:** 10.3390/ani11123413

**Published:** 2021-11-30

**Authors:** Massimo Orioles, Marco Galeotti, Pierpaolo Patarnello, Stefano Pizzolitto, Donatella Volpatti

**Affiliations:** 1 Department of Agricultural, Food, Environmental and Animal Sciences, DI4A, University of Udine, 33100 Udine, Italy; marco.galeotti@uniud.it (M.G.); donatella.volpatti@uniud.it (D.V.); 2 Department of Prevention ASL Le SIAV.B Lecce Sud, 73100 Lecce, Italy; pierpaolo.patarnello@libero.it; 3 Department of Human Anatomic Pathology, Azienda Universitaria Integrata di Udine, University of Udine, 33100 Udine, Italy; stefano.pizzolitto@asufc.sanita.fvg.it

**Keywords:** lipoma, spindle cell lipoma, fibrolipoma, atypical spindle cell-like lipoma, tumors, striped sea bream

## Abstract

**Simple Summary:**

Mesenchymal neoplasms of the skin are common tumors in domestic animals and often reported in fish pathology. Among them, lipomas are frequently observed, but the definition of their different histological features and their classification in fish are still in their primordial stages. In the present work, the authors describe different type of lipomas found in a wild stock of striped seabream and outline their key differential microscopical features. Two new types of lipoma never observed in fish pathology, defined as spindle cell lipoma and atypical spindle cell-like lipoma, are reported.

**Abstract:**

Cutaneous neoplasms affecting wild striped bream (*Lythognathus mormyrus*) have been recorded after their introduction in a marine aquaculture farm in the Adriatic Sea. The tumors were evident on 24% of the reared fish, showing single or multiple nodules, with a diameter ranging between 0.5–4.0 cm. Histologically, all the neoplastic lesions were located in the stratum spongiosum of the dermis and were surrounded by a thin capsule of connective tissue. The tumors were predominantly composed of adipocytes grouped and surrounded by a thin net of fibroblasts and collagen fibers. In some lipomas a mixture of adipocytes and uniform spindle cells were also observed. Fibroblasts and collagen fibers, or spindle cells, showing few mitotic figures were mainly observed in other nodules. Three of the tumors showed bands of cells with elongated nuclei. Five neoplasms differed from the classic spindle cell lipoma due to the presence of scattered giant cells. These cells presented acidophilic abundant cytoplasm with multiple hyperchromatic nuclei showing a concentric “floret-like” arrangement. The tumors were further characterized by ultrastructural observations that allowed ruling out the presence of virus-like particles within the lesions. Histological features of the masses lead to the identification of four prevalent patterns of neoplasms: lipoma, fibrolipoma, spindle cell lipoma (SCL), and atypical spindle cell-like lipoma (ASCL). The different neoplasms could arise from the transformation of mesenchymal cells of dermal origin. To the author’s knowledge, this is the first report describing key differential histological and ultrastructural features of these neoplasms in striped sea bream.

## 1. Introduction

The presence of neoplastic lesions in wild and reared marine fish has been widely described in the literature [1,2,3]. According to the World Health Organization International histological Classification of Neoplasias of Domestic Animals [4], neoplasias arising from adipose tissue are either benign or malignant. Lipoma is considered a benign neoplasia of well-differentiated adipocytes that is found in most domestic animals [5]. Among the neoplasms presented in the Registry of Tumors in Lower Animals of the Smithsonian Institute [6], lipoma and fibrolipoma are the most frequent in bony fishes (5.7%), second only to the fibroma (6.2%), but become the first (4.8%) if we exclude the salmonids where these kind of neoplastic lesions are very uncommon (0.8%). Lipomas commonly arise from mature adipocytes often separated by fibrous septa of varying thickness or containing fibrous areas [7,8]. Typically, the lipoma is a well-differentiated, rounded, encapsulated mass, but can infiltrate other tissue including muscles, being poorly demarcated [1,8]. In domestic animals, the classification of lipoma has been recently defined by Roccabianca et al., 2020 and includes lipoma, fibrolipoma, chondrolipoma, osteolipoma, lipoleyomioma, spindle cell lipoma, and infiltrative lipoma [8]. To date, this classification has not been applied to fish. These type of tumors have been reported from a large variety of fish including carp bream *Abramis brama* (L.) [9], largemouth bass, *Micropterus salmoides* (Lacepede) [10], black crappie, *Pomoxis nigromaculatus* (Lesueur) [6], channel catfish, *Ictalurus punctatus* (Rafinesque) [11], European eel, *Anguilla anguilla* (L.) [12] striped mullet, *Mugil cephalus* (L.) [13], common dab, *Limanda limanda* (L.) [14], northern bluefin tuna, *Thunnus thynnus* (L.), where they appear particularly frequent [15,16,17], striped seabream, *Lithognathus mormyrus* [18], European seabass, *Dicentrarchus labrax* (L.), [19], molly, *Poecilia velifera* (Regan), [20], and koi carp, *Cyprinus carpio* [21]. Angiolipoma has been described in a Siamese fighting fish, *Betta splendens* [22], whereas liposarcomas have rarely been diagnosed in fish [7] but have been reported in Dragonet, *Callionymus lyra* [23] halibut, *Hippoglossus hippoglossus* [24], clownfish, *Amphiprion ocellaris* [25], and flower horn fish, a hybrid cichlid [26].

In human medicine, lipoma is one of the most common benign mesenchymal neoplasms, and liposarcoma is one of the most common malignant mesenchymal tumors [27]. Lipomas can be histologically classified as classic lipoma, fibrolipoma, angiolipoma, intramuscular lipoma, chondrolipoma, chondroid lipoma, sialolipoma, and spindle cell lipoma (SCL) based on the histopathological appearance of the tumor stroma [28]. Histologic spectrum of SCL includes pleomorphic lipoma [29]. This tumor microscopically consists of a mixture of mature adipocytes, bland spindle cells, bundles of ropey collagen, myxoid changes, and blood vessels, and is further characterized by the presence of floret-like giant cells [29].

The aim of the present paper is to describe and outline histological and ultrastructural key features to differentiate lipoma, fibrolipoma, spindle cell lipoma, and its variant here defined atypical spindle cell-like lipoma (ASCL). These cutaneous multiple neoplastic lesions occurred in a group of wild striped bream caught in the Adriatic Sea, transferred to an intensive fish farm located in the south of Italy and sent to the author’s institution for diagnostic purposes. In addition, authors would like to investigate viral aetiology in association with adipose tissue neoplasia, as it has never been reported in fish.

## 2. Materials and Methods

### 2.1. Fish

Five hundred and fifty wild striped bream were introduced in quarantine tanks of a mariculture located in Puglia region (Italy). The mean weight of the subjects was 220 ± 25 g and the rearing density 8 kg/m^3^. The appearance of neoplastic lesion was detected on 24% of the reared fish immediately after their introduction to the quarantine tanks. Thirty subjects showing the cutaneous lesions were euthanized by an overdose of MS-222 (300 mg/L) and sent for diagnostic purposes to University of Udine Veterinary Pathology laboratory. From 13 fish, 16 neoplastic lesions were sampled for the histological and ultrastructural evaluation. Three out of the thirteen fish showed multiple nodules. The nodules were numbered from 1 to 16 for description purposes.

### 2.2. Histology

Sixteen nodules and the contiguous tissues were fixed in 10% buffered formalin for 72 h, automatically dehydrated by immersion in ethanol and xylene (TISBE tissue processor, Diapath, Bergamo, Italy) and embedded in paraffin wax (ParaplastPlus, Diapath, Bergamo, Italy) at 58 °C. Serial 5 μm sections were obtained by a programmable microtome (Reichert-Jung 2050, Reichert Technologies, Buffalo, NY, USA) and then stained with haematoxylin–eosin, Gomori, Masson trichromic and observed by a light microscope (Leica DMRB, Leica, Wetzlar, Germany) equipped with a digital camera (Nikon E200, Nikon, Tokyo, Japan)

### 2.3. Transmission Electron Microscopy

Ultrastructural examination was carried out only on a limited number of specimens. Samples of tissue collected from the nodules n° 6, 10, and 12 were fixed in 2% glutaraldehyde (1 h) and 1% osmium tetroxide (1 h), and included in epoxy resin. Ultrathin sections were obtained with a Reichert-Jung ultramicrotome, then stained with a double coloring system involving 3% uranile acetate (20 min) and lead citrate (10 min). Ultrastructural observations were carried by transmission electron microscope (Philips CM10, Philips, Amsterdam, The Netherlands) equipped with a digital camera (BiosprintM ActiVu, Philips, Amsterdam, The Netherlands).

## 3. Results

### 3.1. Gross Morphology

**Figure 1:**
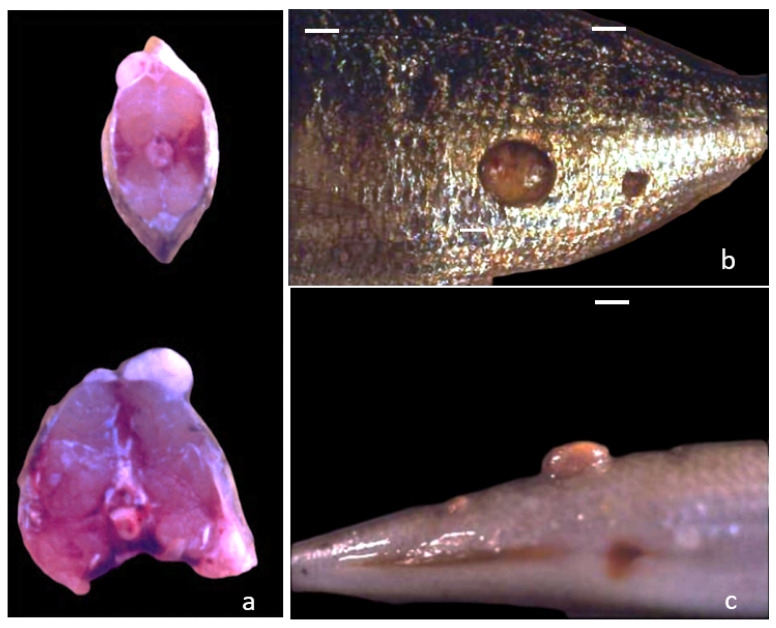
Cut section (**a**) and lateral (**b**) and dorsal (**c**) view of the masses on striped sea bream showing the exophytic growth and a well-circumscribed appearance, mainly present on fish flank. Multiple masses are present on the same fish (bar = 1 cm).

The gross examination allowed detecting the presence of nodular neoformations markedly adherent to the skin and the sub-cutaneous tissue (Figure 1a–c). They were located in several sites of the body, in particular head, flanks, dorsal, and caudal fins. Generally, they were covered by a layer of epithelium, with or without scales. The surface showed dark or pink pigmented aspect (Figure 1b). Nodules were single or multiple in the same subject measuring a diameter ranging between 0.5–4.0 cm (Figure 1b,c). The cut sections displayed homogeneous masses of whitish/greasy tissue, usually well demarcated (Figure 1a). Some nodules showed grey- whitish gelatinous areas in their inner part. Smaller nodules generally had a compact texture, while bigger ones were softer. No macroscopical alterations were detectable in muscle tissue and visceral organs. No parasites were observed nor reported at the farm sites in wet smears and histological samples of skin and gills of all cases.

### 3.2. Histological Features

The histological evaluation allowed observing that all the neoplastic lesions were located in the stratum spongiosum of the dermis and surrounded by a thin capsule of connective tissue. The upper part of epidermis was not involved, except some cases in which its thickness appeared mechanically thinned. Underlying skin layers and muscles were generally unaffected. Histological features of the lesions lead to the identification of four prevalent pattern of neoplasms: lipoma, fibrolipoma, spindle cell lipoma, and atypical spindle cell-like lipoma. Table 1 summarizes the classification of all the collected tumors and their main histological features.

### 3.3. Neoplasms n. 1 to 3: Lipoma

**Figure 2:**
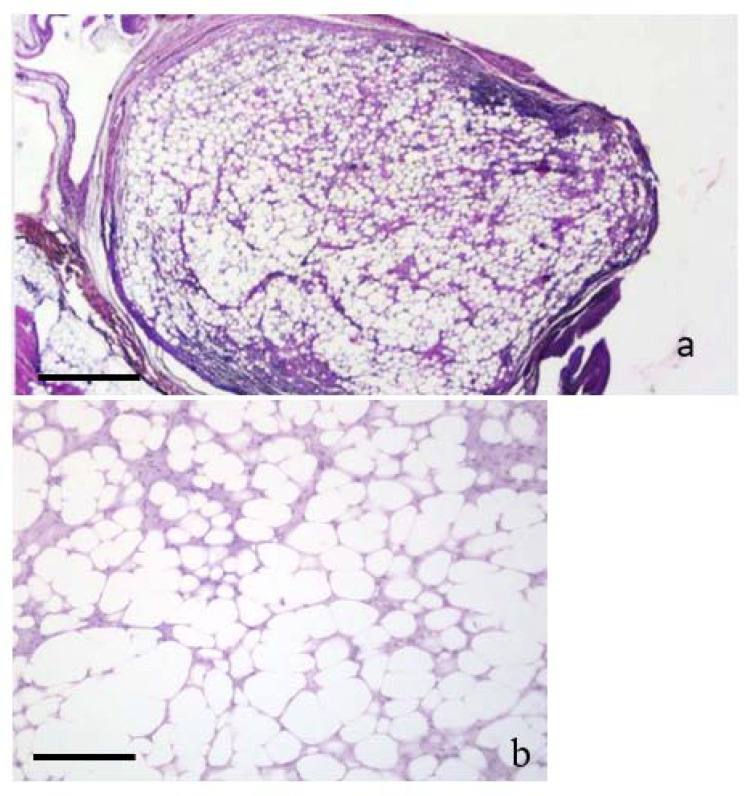
(**a**,**b**) LIPOMA. (**a**,**b**). Histological sections of a lipoma. (**a**) shows the lipoma arising from the dermis. The epidermal layer is mostly unaffected H&E (bar = 500 µm). The capsule is present, and infiltration of lymphocytes can be seen in the subcapsular area. (**b**) Higher magnification of (**a**). The nodule is composed of adipocytes, generally well separated from each other by a thin cytoplasm layer. The large central vacuole displaces the nuclei to the cellular periphery. Cells are almost uniform in size and shape. H&E (bar = 100 µm).

The diameter of these neoplasms ranged from 1 to 8 mm. They were composed of adipocytes showing only small variations in cellular size and shape (Figure 2b). These cells were well differentiated containing a single large clear intracytoplasmatic vacuole and peripheral nuclei, without evidence of mitoses (Figure 2b). Adipocytes were grouped in sheets and surrounded by a thin net of fibroblasts and collagen fibers. The lesions were poorly vascularized (Figure 2b) and a lymphocyte infiltration was evident, especially in the capsule proximity area. No evidence of necrosis was present. These neoplasms were surrounded by a thin connective capsule. They were classified as lipomas.

### 3.4. Neoplasms n. 4 to 8 Fibrolipoma

**Figure 3:**
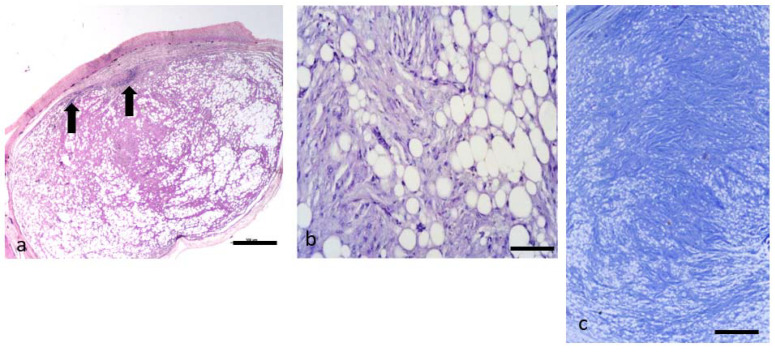
(**a**–**c**). FIBROLIPOMA. (**a**–**c**). Histological section of a fibrolipoma. (**a**) shows the prevalence of fibroblasts and the collagen fibers especially in the center of the neoplasia. Groups of infiltrating adipocytes and adipoblasts are located among the connective component. The capsule is present, and infiltration of lymphocytes (black arrows) is present in the subcapsular area. H&E (bar = 500 µm). (**b**) Higher magnification of the (**a**) showing groups of adipocytes in between the fibroblastic component H&E (bar = 100 µm). (**c**) shows a stroma composed by a mixture of collagenous and reticular fibers which stains blue with Masson’s trichromic and are arranged in wavy bundles. Masson’s stain (bar = 200 µm).

Five neoplasms showed a prevalent composition of fibroblasts and collagen fibers. Small groups of adipocytes and adipoblasts were located among the fibroblastic components (Figure 3a–c). In some nodules these fibroblastic components were prevalent, in others the adipocytes were numerous with fibrous tissue embedded among them (Figure 3a–c). The stroma was composed by a mixture of collagenous and reticular fibers. The former stained blue with Masson’s trichromic and were arranged in wavy bundles (Figure 3c). The reticular fibers consisted of fine argyrophilic filaments detected with silver impregnation. The vascularization of these tumors was inconspicuous. Necrotic tissue was not evident, also in the largest nodules. The capsule was thin and often infiltrated by lymphocytes (Figure 3a arrows); these cells often invaded the internal part of neoplastic parenchyma starting from the capsule, or could be easily detected in proximity to the vessels. In tumor n. 8 there was a prevalent part of star-shaped cells, separated by abundant intercellular matrix, that gave a myxomatous aspect to this tumor. It was possible to identify rare mitotic figures. A definitive diagnosis of fibrolipoma was based on the presence of a variable amount of mature connective tissue with embedded adipocytes.

### 3.5. Neoplasms from n. 9 to 11: Spindle Cell Lipoma

**Figure 4:**
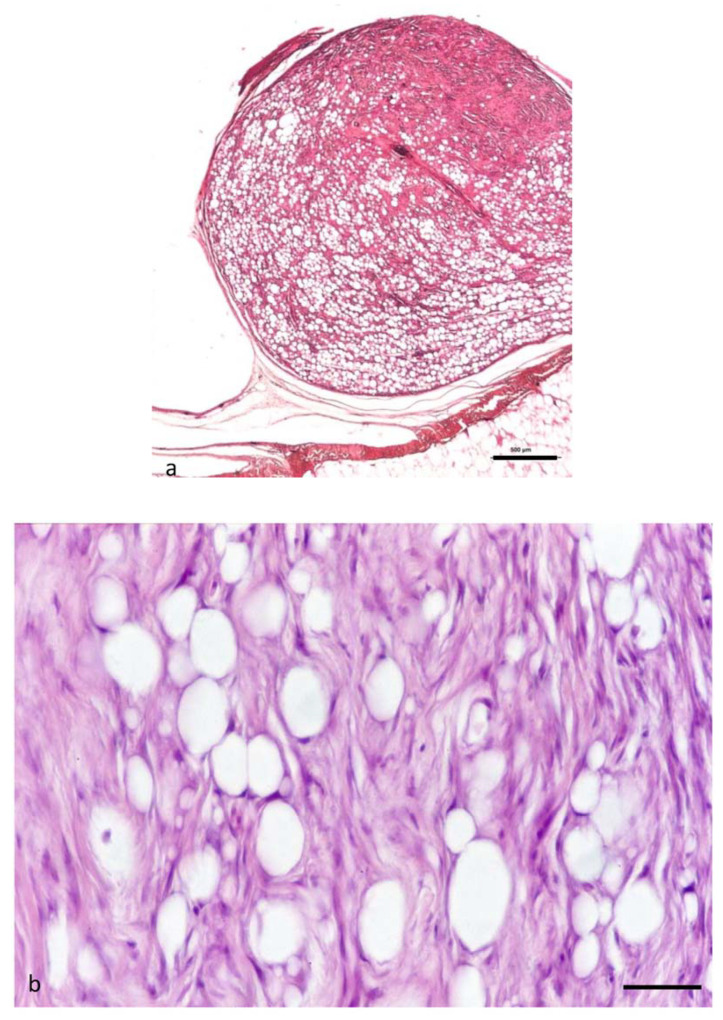
(**a**,**b**) SPINDLE CELL LIPOMA (SCL). (**a**,**b**). Histological section of a spindle cells lipoma. (**a**) shows a net of fibroblasts and collagen fibers surrounding mature adipocytes. Spindle cells are often located in one side of the nodule, while the other side reveals mainly adipocytes. H&E (bar = 500 µm). (**b**) shows how spindle cells tend to be well aligned or sometimes assuming an irregular distribution pattern. These cells tend to have a mild cellular pleomorphism. H&E (bar = 50 µm).

The diameter of these nodules was about 1 mm. They were composed of a mixture of adipocytes and haphazardly arranged bland spindle cells that were closely associated with a variable number of thin, angular collagen fibers. Two of them showed mostly the feature of lipoma, with only a portion of the lesion being replaced by a typical mixture of spindle cells and adipocytes (Figure 4a). Spindle cells were mostly uniform and bipolar, with scant cytoplasm that rarely contained small lipid vacuoles. In some cases, they were assuming an irregular distribution pattern with slight cellular pleomorphism (Figure 4b). Mitoses were rare; they were often located in one side of the nodule, while the other side revealed mainly adipocytes (Figure 4a). In 1 out of 3 of these tumors, adipocytes were rare and the spindle cells presence dominated the histological section of the nodule. The vascular pattern was generally inconspicuous and consisted of few thick-walled vessels of small or intermediate size. In this latter tumor the prevalent cells showed an elongated shape with a “cigar-shaped” nucleus and eosinophilic cytoplasm. These cells, resembling myoepithelial-like cells, were scattered within mature adipose tissue and fibroblasts. All the lesions were surrounded by a thin connective capsule, that in two cases showed an infiltrate composed of mononuclear inflammatory cells, also invading the internal part of the nodule. The microscopic appearance of these three tumors allowed defining them as spindle cell lipomas.

### 3.6. Neoplasms from n. 12 to 16: Atypical Spindle Cell-like Lipoma (ASCL)

**Figure 5:**
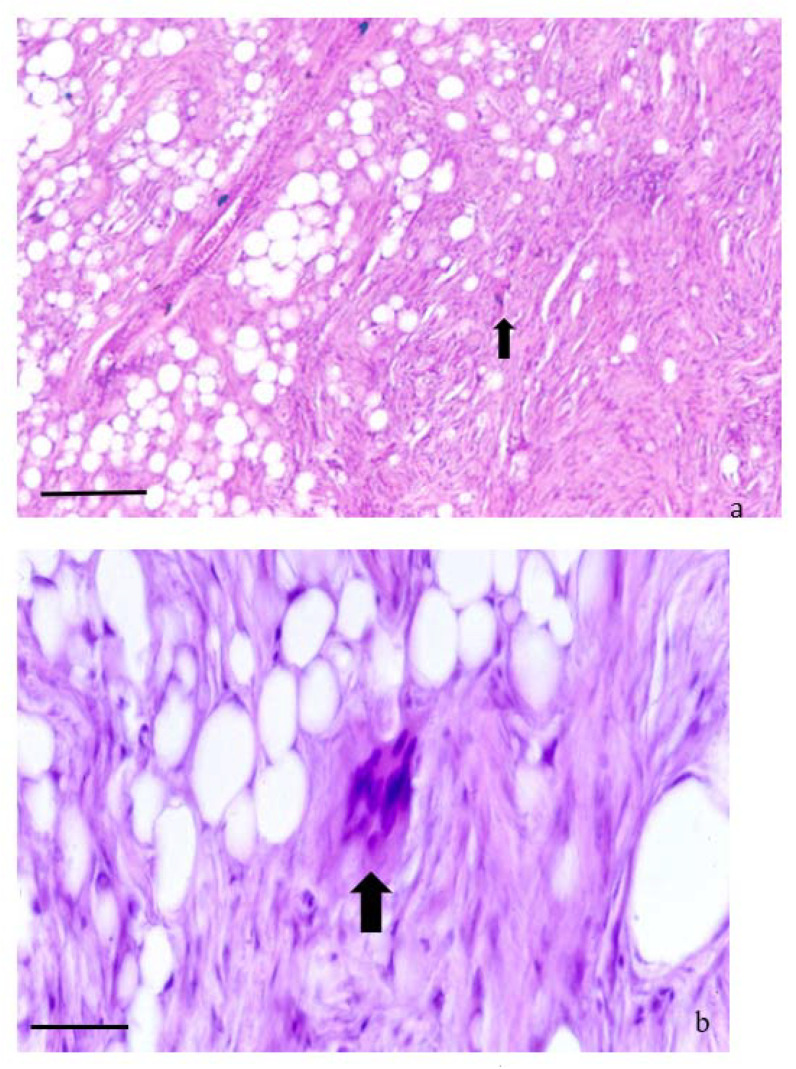
(**a**,**b**). ATYPICAL SPINDLE CELL-LIKE LIPOMA (ASCL). (**a**,**b**). Histological section of an atypical spindle cell-like lipoma. These neoplasms display a prevalent aspect of spindle cell lipoma, for the abundance of spindle-shaped and fibroblastic components. The structure consists of poorly defined whorls and interlacing bundles of fibroblasts (**b**) H&E (bar = 100 µm). (**b**) Higher magnification of the (**a**). The image shows a mixture of adipocytes and uniform spindle cells. These are associated with bundles of collagen. These tumors differ from the spindle-shaped lipoma due to the presence of scattered bizarre multinucleated giant cells (black arrow) H&E (bar = 50 µm).

Five neoplasms displayed a prevalent aspect of spindle cell lipoma, due to the abundance of spindle-shaped and fibroblastic components (Figure 5a,b). The tumors structure consisted of poorly defined whorls and interlacing bundles of fibroblasts (Figure 5a), but they differed from the spindle-shaped lipoma described before due to the presence of scattered bizarre multinucleated giant cells (Figure 5b). These cells presented acidophilic abundant cytoplasm with multiple hyperchromatic nuclei and could resemble the “floret-like” giant cells. Multinucleated giant cells with large, irregular nuclei containing irregular and expanded nucleoli were also frequently observed. Mitotic figures were rare. Another feature of these neoplasms, if compared with the previous, was the relevant vascularization. Moreover, it was possible to observe an infiltration of lymphocytes, especially concentrated in the capsule surrounding the tumor, around the vessels or among the neoplastic cells. These tumors were defined atypical spindle cell-like lipoma (ASCL).

### 3.7. Transmission Electron Microscopy

**Figure 6:**
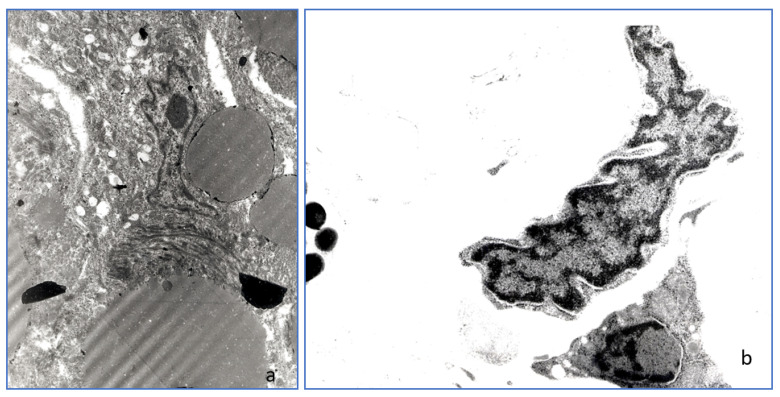
(**a**,**b**) Tumor n. 6 (SPINDLE CELL LIPOMA). (**a**) Lipoblasts with intracytoplasmatic lipidic droplets of various size (12,000×), (**b**) Spindle cells: the nucleus presents folded and corrugate profile (8700×).

**Figure 7:**
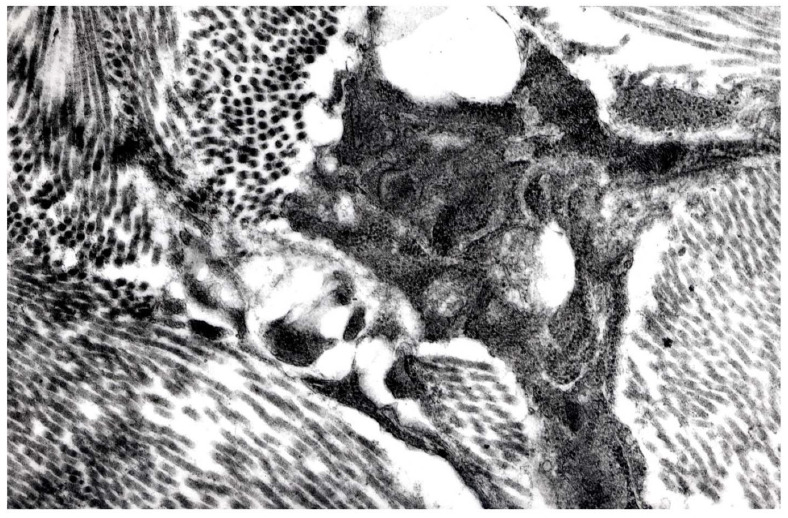
Tumor n. 12 (ATYPICAL SPINDLE CELL-LIKE LIPOMA). Pleomorphic cells with irregular borders within abundant collagen matrix (14,000×).

Ultrastructural findings of the nodules number 6, 10, 12 were rather heterogeneous and consisted of lipoblasts and adipocytes with variably sized intracytoplasmic lipidic droplets, elongated spindle cells with “carinated” and folded nuclei and pleomorphic cells with irregular borders (Figure 6a,b and Figure 7). Cells were embedded within a more or less abundant matrix made up of haphazardly intersected collagen fibers. Some cells showed rare patrimonial neurosensorial bodies or incomplete melanosomes. No evidence of viral particles was found.

## 4. Discussion

Tumors arising from adipose tissue in domestic animals are classified as either benign or malignant [5]. Lipoma is characterized by the presence of well-differentiated adipocytes and can rarely contain collagen, bone tissue, cartilage, smooth muscle, and clusters of small blood vessels [5,8]. Spindle cell lipoma is a rare variant composed by a mixture of adipocytes and haphazardly arranged bland spindle cells forming an expansile mass [8]. To date, lipomas are frequently described in teleost fish of many orders, whereas this is not the case for their malignant counterpart [15,25,26]. Lester et al. [15] reported the incidence of lipoma could be around 1:10,000 fish based on findings at a processing plant. In our study the incidence seems to reach considerable levels, up to 24% of the reared fish. Following the criteria applied recently in domestic animals [8], the authors described here multiple mesenchymal tumors, and classified them as lipoma, fibrolipoma, and SCL. The atypical spindle cell-like lipoma variant of SCL seems to have histological features similar to pleomorphic spindle cell lipoma reported in humans [30]. Therefore, if compared with previous bibliographic descriptions, the present study recognizes two new subtypes of lipomas in fish: SCL and its subtype ASCL.

The lipomas previously described in teleost fish literature are often associated with dermis or hypodermis, mostly visible as skin growths. In some cases, they are reported to infiltrate also muscle tissue [11,17,19]. Different localizations are possible; as an example, Marino et al. [16] described a single lipoma in the dorsal epiaxial musculature above vertebral column in a farmed northern bluefin tuna, *Thunnus thynnus* (L.). In our study all growths are exophytic, they seem to arise only from dermis, and no muscle infiltration or neoplastic spread in other organs are observed. As the cases reported here, flank and dorsal areas of the fish are the main localization reported in the literature, and lipomas are often multicentric [16,18,20]. The presence of a capsule or pseudo-capsule is variably reported in the literature and it does not seem to be usually present in infiltrative lipoma [17]. In our work, a capsule has been observed consistently in all cases without any evidence of infiltration. The term ‘’infiltrative lipoma’’, used in domestic animals and fish pathology, can be slightly misleading as it can be linked with the idea of a certain malignancy; in reality, infiltrative lipomas are considered to be benign as well, with no metastatic potential [8]. Lipomas and liposarcomas in fish can grow variably to enormous size (up to 20 kg [11]) and can result in cutaneous ulcerations [18] leading to complications such as secondary infections [21]. In our cases, epidermis have not been overtly affected, if we exclude a mild thickening in two cases. Although multiple lipomas in wild striped seabream have been reported in the literature [18], the authors believe the present case series may be particularly interesting as it shows a variety of different neoplasms in the same stock of wild fish and illustrates their key histological features. The tumors described here were detectable as single or multiple lesions and sometimes nodules showing different histological features were present on the same individual fish; for example, a SCL and ASCL, or two SCL and one ASCL, respectively.

The term spindle cell lipoma was used by the authors to define a neoplasm which is composed of elongated cells, spindle shaped, resembling those observed in domestic animals [8]. The peculiar arrangement of spindle cells within the nodules can be observed also in human tumors [30]. The spindle cells are mainly located in one side of the nodule, while the other side reveals adipocytes only. Mitoses can be rare, while the presence of myxoid matrix is not uncommon [8]. A more recent and specific histological classification in human medicine (low-fat and fat-rich lipoma) is based on the relative proportions of spindle cells and adipocytes [28]; to date, this classification has no counterpart in domestic animals [8].

In human medicine, several variants of SCL have been reported and the key to their histopathological diagnosis is the identification of the spindle cell population. Immunohistochemical (IHC) analysis of SCL allows further characterizing these tumors in humans. Common features are a strong positivity for CD34 of spindle cells, positivity for S100 of mature adipocytes, positivity for mast cells (MC) tryptase and negativity to retinoblastoma protein (pRb) of spindle cells [27,30]. Although immunohistochemistry and electron microscopy approaches have also been applied to teleost oncology to date [31,32], the characterization of different types of tumors and some clinical correlations through these techniques are still in their primordial stages. To the best of our knowledge, IHC has never been performed on lipoma neoplasms in fish species. The definition of SCL in domestic animals can be based on hematoxylin-eosin morphology [8]. In SCL, mitoses are rare, and spindle cells are haphazardly arranged and have a bland appearance with low cell proliferation; rare lipid vacuoles can be seen in the intracellular space. As described by the authors in this study, thinner collagen fibers and a more angular appearance than those seen in fibrolipoma seem to be one of the key features when differentiating these two tumors [8].

The nomenclature ASCL we included in the present description originates from the similarity of some cellular elements with those frequently detectable in human medicine in spindle cell pleomorphic lipoma [30]. These cells are often named “floret cells”, due to the characteristic arrangement of nuclei. In comparison with human spindle cell pleomorphic lipoma, the fish neoplasms seem to be characterized by more abundant connective and fibrocellular structure, mingled with adipocytes [30]. For this reason, and the presence of spindle cells, the definition of atypical spindle cells such as lipoma seems to better describe the features of the nodules under investigation. It is worth considering that the presence of relevant vascularization seems to be a peculiar feature of these neoplasms in our study, when compared with the other type of lipoma observed, especially with fibrolipoma. No evidence of vascular or lymphatic vessels involvement by tumoral cells was detected, which gives an idea of the local and benign nature of these tumors. The abundance of vascularization can be found in angiolipoma and angioleiomyoma reports [26,33]. In our cases, the absence of blood vessel involvement in neoplastic transformation was considered a differential feature with angiolipoma.

Other studies in fish have reported similar structures resembling fibrolipoma. The lipomas reported previously in Southern bluefin tuna (*T. maccoyii*) by Lester and Kelly (1983) [15] and in striped seabream by Gomez (2009) [18] appear to contain irregular bands of connective tissue. However, no floret cells were described, and vascularization always seemed scarce. A peculiar, but not surprising, aspect of the neoplasm described in this study is the constant presence of inflammatory infiltration especially in the subcapsular region. This seems to be composed mainly by mononuclear inflammatory cells, especially lymphocytes. The possible presence of inflammatory infiltrate in lipomas is reported in domestic animals [8], where numerous foamy macrophages can be detected within the lesion [5]. In fish literature, only Gomez (2009) [18] described the presence of melanomacrophages aggregates possibly related to the tumoral process, but no inflammatory infiltration has been reported.

Several hypotheses have been considered about the possible etiopathogenesis of lipoma, but at present conclusions are still scarce and inconsistent. Bony dysraphism, when the lipoma develops close to bone and near the skull or vertebral column [17], errors in fat metabolism [19], endocrine, or neurologic disorders, and exposure to chemicals, such as N-methyl-N′-nitro-N-nitrosoguanidine [34] have been proposed. For the fish under study, as most others, the underlying etiology of the lipoma is unknown and warrants further investigation. Basic water parameters were generally normal, and no relevant parasites were detected. The high incidence in the same farm might have been caused by chemical water contamination where fish were originally caught, but this hypothesis could not be confirmed by water analysis data. Although lipomas inducted by viruses have never been reported [35] presence of viral particles have been ruled out with the use of electronic microscopy.

## 5. Conclusions

This research allowed us to describe the presence of multiple neoplasms displaying several histological features, at any rate due to the neoplastic alteration of mesenchymal cells of dermal origin. The present study proposed two new definitions of teleost lipomas (SCL and ASCL) and described key elements for their recognition. Future works, also based on IHC analysis, are needed to further characterize these tumors in fish, to add some more clinical and prognostic values, and to study the possible etiology.

## Figures and Tables

**Table 1:** Histological classification of the 16 neoplasms collected from striped sea bream and relative differential features.

Number	Histological Classification	Histological Features
**1–3**	**Lipoma**	**Prevalent features:** Mature well-differentiated adipocytes with a thin net of fibroblasts. Negligible variation in cell size and shape. **Vascularization:** Poor **Inflammatory cells infiltration:** Mainly lymphocytes in proximity to capsular area
**4–8**	**Fibrolipoma**	**Prevalent features:** Prevalent fibroblasts and collagen fibers with small groups of adipocytes and adipoblasts. **Vascularization:** Poor **Inflammatory cells infiltration:** Lymphocytes infiltrating the neoplastic parenchyma from the capsule
**9–11**	**Spindle cell lipoma (SCL)**	**Prevalent features:** Mature adipocytes and spindle cells associated with a variable number of angular collagen fibers. **Vascularization:** Inconspicuous; it consists of few thick-walled vessels of small or intermediate size **Inflammatory cells infiltration:** Mononuclear cells infiltrating all areas of the nodule.
**12–16**	**Atypical spindle cell-like lipoma (ASCL)**	**Prevalent features:** Spindle cells but presence of scattered multinucleated giant cells resembling “floret-like” giant cells with acidophilic abundant cytoplasm and multiple hyperchromatic nuclei. **Vascularization:** Relevant **Inflammatory cells infiltration:** Diffuse lymphocytes infiltration around vessels, underneath the capsule and in between neoplastic cells.

## Data Availability

The data presented in this study are available upon request from the corresponding author.
